# One-stage freehand minimally invasive pedicle screw fixation combined with mini-access surgery through OLIF approach for the treatment of lumbar tuberculosis

**DOI:** 10.1186/s13018-022-03130-4

**Published:** 2022-04-19

**Authors:** Wenshuai Fan, Guangling Yang, Tianyao Zhou, Yanchao Chen, Zhenchao Gao, Weili Zhou, Yutong Gu

**Affiliations:** 1 Department of Orthopaedics, Ruijin Hospital, Shanghai Jiao Tong University School of Medicine, Shanghai, 200025 China; 2 Department of Orthopaedic Surgery, Shanghai Public Health Clinical Center, Fudan University, Shanghai, 201508 China; 3 Department of Orthopaedic Surgery, Zhongshan Hospital Fudan University, Shanghai, 200032 China

**Keywords:** Lumbar tuberculosis, Minimally invasive spine surgery, Pedicle screw fixation, OLIF, Debridement, Bone graft fusion

## Abstract

**Objective:**

To compare one-stage freehand minimally invasive pedicle screw fixation (freehand MIPS) combined with mini-access surgery through OLIF approach with posterior approach for treatment of lumbar tuberculosis (TB), and evaluate its feasibility, efficacy and safety in debridement, bone graft fusion and internal fixation.

**Methods:**

48 patients with single segment lumbar TB from June 2014 to June 2017 were included. Among them, 22 patients underwent one-stage freehand MIPS combined with mini-access surgery through OLIF approach (group 1), 26 patients were treated with posterior open surgery (group 2). Duration of operation, blood loss, and stay time in hospital were compared. Pre- and postoperative visual analog scale (VAS) pain scores, Oswestry disability index (ODI), erythrocyte sedimentation rate, complications and images were also recorded.

**Results:**

Patients in group 1 showed significantly less blood loss (165 ± 73 ml vs 873 ± 318 ml, *P* < 0.001), shorter stay time in hospital (6/4–8 days vs 12/8–15 days, *P* < 0.001), while longer duration of operation (185 ± 14 min vs 171 ± 12 min, *P* < 0.001) than group 2 did. VAS scores significantly decreased after surgery in both groups, however, VAS scores of group 1 were significantly lower than that of group 2 immediately after surgery and during follow-ups (*P* < 0.001). ODI of group 1 was also significantly lower than that of group 2 at 12-month after surgery (*P* < 0.001).

**Conclusion:**

One-stage freehand MIPS combined with mini-access surgery through OLIF approach is a feasible, efficient and safe method in treating single segment lumbar TB. It shows advantages of less surgical trauma and faster postoperative recovery.

## Introduction

Spinal tuberculosis (TB) is the most common extrapulmonary TB, accounting for about half of osteoarticular TB [1]. The treatment of spinal TB is still a challenging. Although tuberculostatics is the cornerstone for treating TB, patients with spinal instability, neurologic compression, spinal deformation or poor response to conservative treatment need surgical intervention. Debridement also shows a vital role for patients with abscess formation [2–5]. The surgical strategies of spinal TB mainly include anterior, anterior combined posterior, and posterior approach for debridement, decompression, bone graft fusion and internal fixation [6]. These approaches show their own advantages and disadvantages, the choice for optimal surgical method remains controversial [7, 8].

With advancements in surgical techniques and technologies, minimally invasive surgeries provide a new option for the treatment of spinal TB [9, 10]. Freehand minimally invasive pedicle screw fixation (freehand MIPS) is our previous established minimally invasive technique of internal fixation, which does not require traditional large posterior incision, extensive dissection and traction of the paravertebral muscle [11]. Oblique lateral interbody fusion (OLIF) is a minimally invasive technique through anterior-lateral approach, which has showed confirmed clinical effects in the treatment of lumbar degenerative diseases [12, 13]. However, the efficacy of this approach in treating lumbar TB still needs further evaluation. In this study, one-stage freehand MIPS combined with mini-access surgery through OLIF approach was used to treat single segment lumbar TB and compared with posterior open surgery to evaluate its feasibility, efficacy and safety.

## Materials and methods

### Subjects

**Fig. 1:**
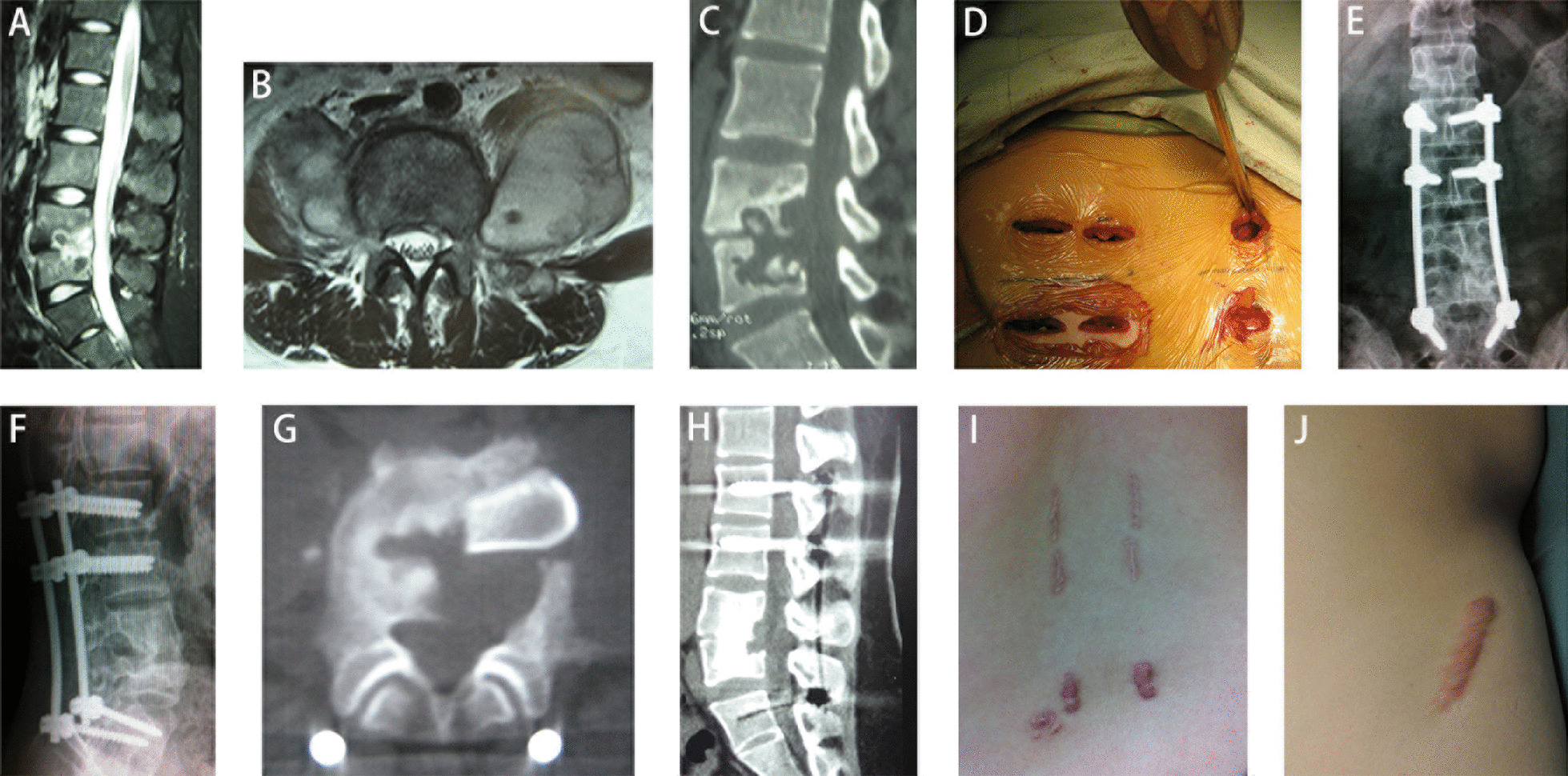
A 20-year male patient with L4–L5 tuberculosis accompany with psoas abscesses. Preoperative MRI (**A**, **B**) and CT (**C**) showed destruction of L4–L5 and psoas abscesses. Freehand MIPS (**D**). Postoperative x-ray (**E**, **F**) and CT (**G**) showed good location of the autogenous iliac bone and internal fixation. CT at 12-month after surgery (**H**) showed bone fusion achieved without internal fixation failure. Surgical incisions of freehand MIPS (**I**) and OLIF (**J**)

**Fig. 2:**
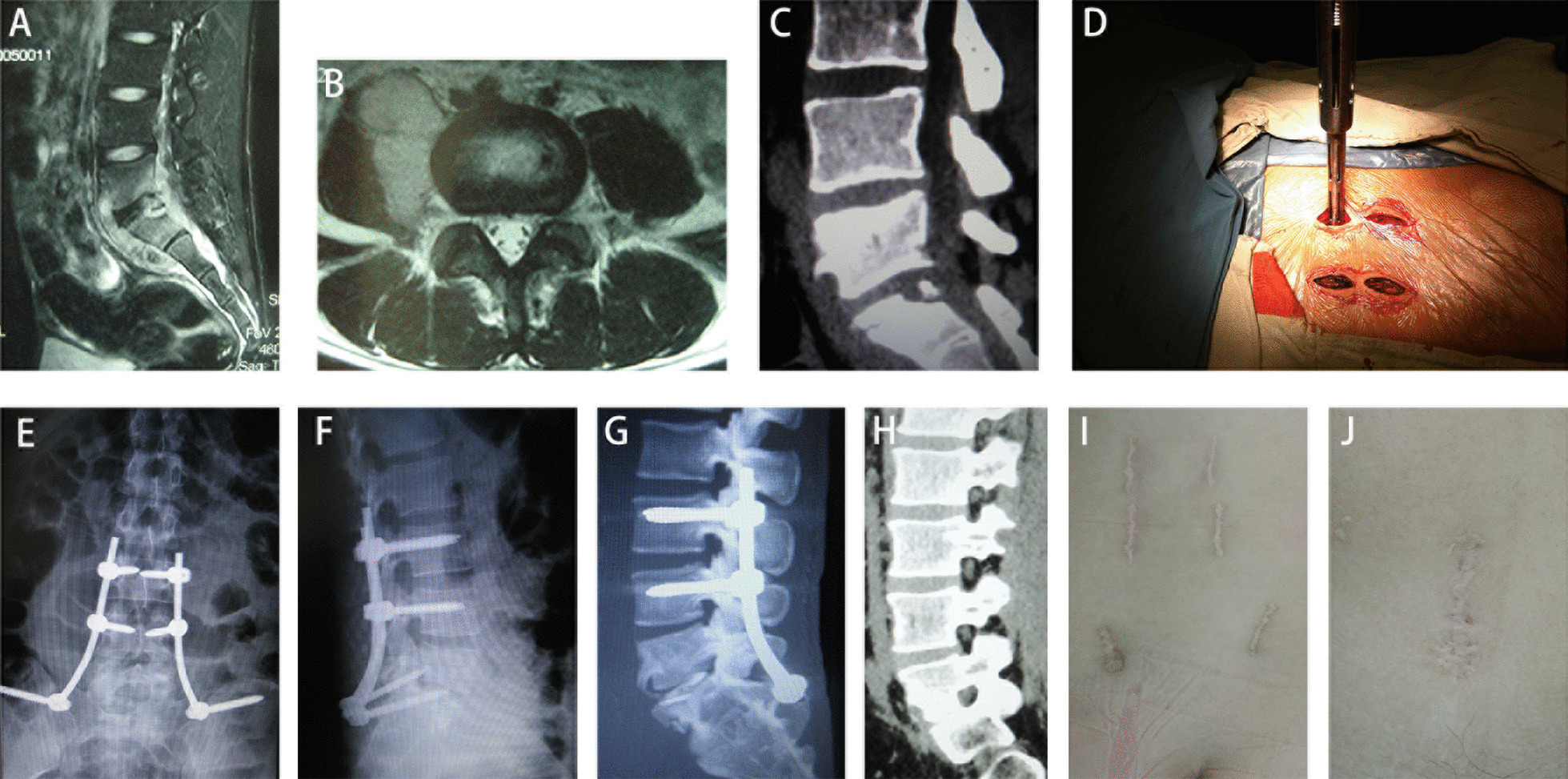
A 20-year male patient with L5–S1 tuberculosis accompany with psoas abscesses. Preoperative MRI (**A**, **B**) and CT (**C**) showed destruction of L5–S1 and psoas abscesses. Freehand MIPS and minimally invasive iliac screws (**D**). Postoperative x-ray (**E**, **F**) and CT (**G**) showed good position of the autogenous iliac bone and internal fixation. CT at 12-month after surgery (**H**) showed bone fusion achieved. Surgical incisions of freehand MIPS and iliac screws fixation (**I**) and OLIF (**J**)

We retrospectively reviewed the medical records of lumbar TB patients who underwent surgical treatment from June 2014 to June 2017. Inclusion criteria: (1) patients with persistent low back pain, with no alleviation after anti-TB therapy, possibly accompanied with TB toxemia, such as lassitude, afternoon pyrexia, night sweats, but without neurological symptoms; (2) diagnosed as single segment lumbar TB by radiology, with spine instability (no continuous trabecular bone between adjacent vertebrae), massive bone defects, or abscess formation, without neurological compression and kyphosis (Figs. 1A–C, 2A–C); (3) undergone operation by one-stage freehand MIPS combined with mini-access surgery through OLIF approach or posterior open approach; (4) follow-up more than 1 year. Patients with a congenital spinal deformity or lumbar surgery before were excluded. This study was approved by the medical ethical committee of Zhongshan hospital Fudan University.

### Surgical procedures

#### Group 1: freehand MIPS + mini-access surgery through OLIF approach

*Freehand MIPS* Patients were placed in a prone position. After perspective positioning, 1 cm incisions were made in back 3 cm away from the midline (Wiltse approach). Above and below the involved vertebra, the adjacent 1 or 2-level lumbar vertebrae’s superior articular facet and root of transverse process or posterior superior iliac spine were exposed through multifidus interspace. The transverse process of T11, T12 or superior articular facet of S1 were also exposed if necessary. Under direct vision, the entry site was located at the bisecting midline of the transverse process for T11, T12 pedicles, the junction between the lateral border of the superior articular facet and bisecting midline of the transverse process for lumbar pedicles or around posterior superior iliac spine for iliac. Once the pedicle or iliac had been identified, a handheld curette was used to enter the pedicle or iliac and 4 pairs of pedicle screws or 2 iliac screws of appropriate length are then introduced into the vertebral body or iliac after the integrity was verified in four quadrants to ensure that a solid tube of bone exists (Figs. 1D, 2D). Two rods were contoured according to the normal spine curve and placed over the pedicle screws through subcutaneous soft tissues (Figs. 1E–F, 2E–F).

*Mini-access surgery through OLIF approach* Adjust patients in a lateral position (L1–L5) or oblique position (L5–S1) on their right side. A 4 cm skin incision was made along the anterior line of marked disc. Blunt dissection into the retroperitoneal space along the direction of external, internal, and transverse abdominal muscles and pushing the peritoneum anteriorly to expose psoas. The lesion in L1–L5 was access through the interval between abdominal vessels and psoas. If there was no abscess, expose the lesion by peeling psoas major muscle back from the front; if there was an abscess along psoas major muscle, enter the lesion through the abscess. The lesion in L5–S1 could be reached through the approach between both iliac vessels. The abscess and necrotic tissue were completely removed under direct vision. Then cortical iliac bone was grafted according to the height of intervertebral space or length of vertebral slot (Figs. 1G, 2G). Streptomycin was local applied and one negative pressure drainage tube was placed before closing the incision.

#### Group 2: posterior open surgery

After general anesthesia, patients were placed in a prone position. A midline incision was made to elevate subperiosteally the paraspinal muscle and expose the spinal process, bilateral lamina and facet joints. Pedicle screws were inserted into the adjacent 2-level vertebrae above and below the involved vertebra. The dural sac was exposed following removal of spinal process and lamina. After the facet joint and pedicle were removed, the abscess and necrotic tissue were reached and debrided, then suitable cortical iliac bone was grafted. Two rods were fixed over the pedicle screws. Streptomycin was applied locally and a drainage tube was placed before suturing the incision.

### Postoperative care

Patients continued to received anti-TB treatments postoperatively and prevent infection. When the drainage volume was less than 20 ml/24 h, the tube was removed. Then patients were instructed to wear orthosis to get out of bed and perform rehabilitation exercises. Patients without contraindication were encouraged to remove the internal fixation through the original minimal incision 1 year after surgery with bone fusion reaching to Eck grade I [14].

### Outcome indexes

Clinical outcomes: (1) operative time, blood loss and postoperative hospital stay; (2) visual analogue scale (VAS) score before and immediately, 1, 2, 3, 6, 12-month after surgery; (3) Oswestry disability index (ODI) before and 12-month after surgery; (4) erythrocyte sedimentation rate (ESR), liver and renal function every 3 months after surgery; (5) complications, such as incision infection, internal fixation or bone graft failure.

*Imaging outcomes* anteroposterior and lateral x-ray and CT were taken immediately, 3, 6, 12-month postoperatively to evaluate bone fusion according to Eck fusion grading system [14].

### Statistical analysis

SPSS 25 software (SPSS Inc., Chicago, IL, USA) was used to perform statistical analysis. *Student* t test was used for comparison of quantitative data, including age, duration of operation, blood loss, stay time in hospital, follow-up period, VAS, ODI and ESR. *Chi-square test* was used for comparison of qualitative data, including gender and lumbar TB level. *P* < *0.05* was considered to be a significant difference.

## Results

Clinical data is summarized in Table 1. In group 1, 22 patients were treated with one-stage freehand MIPS combined with mini-access surgery through OLIF approach. In group 2, 26 patients obtained posterior open surgery. There were no significant differences in age, gender and lumbar TB level between the two groups. The average operation time of group 1 was 185 ± 14 min while that of group 2 was 171 ± 12 min (*P* < 0.001). The average blood loss of group 1 was 165 ± 73 ml while that of group 2 was 873 ± 318 ml (*P* < 0.001). The average stay time in hospital of group 1 was 6 (4–8) days while that of group 2 was 12 (8–15) days (*P* < 0.001).

**Table 1:** Comparison of clinical data between groups 1 and 2

	Group 1	Group 2	P value
Age (years)	53 ± 16	54 ± 16	0.686
Gender			
Female	8	8	0.682
Male	14	18	
Level			
L1–L2	4	5	0.998
L2–L3	3	4	
L3–L4	5	5	
L4–L5	8	10	
L5–S1	2	2	
Duration of operation (min)	185 ± 14	171 ± 12	0.000
Blood loss (ml)	165 ± 73	873 ± 318	0.000
Stay at hospital (days)	6(4–8)	12(8–15)	0.000
Follow-up period (months)	20 ± 2	20 ± 2	0.839

The average follow-up time was 20 ± 2 months in two groups. Respectively, the mean preoperative VAS scores were 8 (6–10) and 8 (7–10) in group 1 and 2, obviously decreased to 3 (2–5) and 6 (5–7) immediately after surgery, further decreased to 1 (0–2) and 3 (2–4) at 1-month after surgery, and basically maintained at this level until the last follow-up. The mean VAS scores in group 1 were significantly lower than that of group 2 at any time points after surgery (*P* < 0.001) (Table 2). The mean preoperative ODI were 41.6 ± 10.1 and 43.4 ± 9.3 in group 1 and 2, respectively. The mean ODI significantly decreased in both groups and ODI of group 1 was significantly lower than that of group 2 at 12-month after surgery (*P* < 0.001) (Table 3). ESR of all patients decreased gradually after surgery and returned to normal levels within 3 months. No difference of ESR was found between the two groups (Table 4). At 12-month after surgery, all patients obtained bone fusion of grade I, without internal fixation loosened or broken and abscess reoccurrence of the surgical site (Figs. 1H, 2H). The internal fixation was removed in 12 cases of group 1 while 6 cases of group 2. In group 2, there were 2 cases of dural sac tear, 3 cases of leg numb and hypoesthesia and 1 case of leg muscle weakness.

**Table 2:** VAS pain assessments of two groups

Group	Preoperative	Postoperative	1-month	2-month	3-month	6-month	12-month
1	8 (6–10)	3 (2–5)	1 (0–2)	1 (0–2)	1 (0–2)	1 (0–2)	1 (0–2)
2	8 (7–10)	6 (5–7)	3 (2–4)	3 (2–4)	2 (2–3)	3 (2–4)	3 (2–4)
*P* value	0.257	0.000	0.000	0.000	0.000	0.000	0.000

**Table 3:** ODI of two groups (%)

Group	Preoperative	12-month
1	41.6 ± 10.1	9.2 ± 2.9
2	43.4 ± 9.3	15.3 ± 4.1
*P* value	0.527	0.000

**Table 4:** ESR of two groups (mm/H)

Group	Preoperative	Postoperative	3-month	6-month	12-month
1	63.7 ± 25.3	45.6 ± 13.9	19.7 ± 6.9	12.5 ± 3.2	11.6 ± 2.2
2	60.3 ± 25.6	46.3 ± 15.5	22.6 ± 6.8	13.3 ± 3.7	12.7 ± 2.8
*P* value	0.649	0.875	0.145	0.407	0.140

## Discussion

Lumbar is the most common site of spinal TB due to its maximum mobility and weight-bearing. There are various surgical methods for lumbar TB, mainly including anterior, anterior combined posterior, and posterior surgery [15]. Anterior surgery has advantages in sufficient lesion debridement under a direct vision, but with a high risk of viscera or vascular injury and instrument failure [16]. Posterior surgery shows advantages in deformity correction and stability reconstruction, but with a controversy of thorough lesion debridement and ideal anterior column bone graft [17, 18]. Anterior combined posterior surgery could achieve radical debridement and strong internal fixation, but with more surgical trauma and longer postoperative recovery [19]. There is still debate in surgical approach for lumbar TB until now.

Here, one-stage freehand MIPS combined with mini-access surgery through OLIF approach were used as a novel strategy for intervention of lumbar TB. Freehand MIPS implants pedicle screw through minimal-access in a Wiltse approach under direct vision and fixing rods through subcutaneous soft tissues and muscles [20]. Different from traditional midline incision, the Wiltse approach provides a more direct approach to the transverse processes and pedicles, protecting attachment of muscle to bone, avoiding disruption of supraspinous and interspinous ligaments. Compared to percutaneous pedicle screws, freehand MIPS uses incisions of similar size but with easier manipulation and less fluoroscopy during the operation [21–24].

Mini-access surgery through OLIF approach is performed along the direction of external, internal, and transverse abdominal muscle fibers and fully utilizes the retroperitoneal intermuscular space between aorta and psoas for L1–5 or between bilateral iliac vessels for L5–S1, making it possible to directly reach the lesion and perform debridement under direct vision, reducing the risk of vessel or neurologic injury. In ALIF (Anterior Lumbar Interbody Fusion) for L5–S1, rectus sheath could be damaged. OLIF approach could move peritoneal contents to the opposite side with the help of gravity, making anatomical structures revealing easily, especially as compared to anterior approach in ALIF for L5–S1 [25]. When there is no psoas abscess, OLIF exposes the lesion of L1–5 by peeling psoas back to avoid damaging the nerves in psoas while open anterior-lateral surgery usually via iliopsoas may damage these nerves [5]. One-stage freehand MIPS combined with mini-access surgery through OLIF approach operated in a manner similar to traditional anterior combined posterior surgery, however, combined application of these two minimally invasive techniques had almost no damage to paraspinal muscles and no need to enter spinal canal [26]. The results in our study showed that patients receiving this surgery had less blood loss, shorter stay time in hospital, better pain relief and ODI during the follow-up than those receiving posterior open surgery. And shorter scar was saw in these patients receiving minimal invasive surgery (Figs. 1I–J, 2I–J).

Reconstruction of spinal stability is important in determining the outcome of spine surgery after debridement. According to the theory of three column spine [27], anterior and middle column are main structures for support of vertebral bodies, however, anterior and middle column are undermined in most spinal TB cases [28]. To strengthen the biomechanical stability of spine, freehand MIPS was achieved by Wiltse approach without undermining the structure of posterior column. Our previous studies reported that freehand MIPS enable adequate internal fixation in lumbar fracture or metastatic tumor [21–24]. Meanwhile, autogenous cortical iliac bone was grafted to the intervertebral space or vertebral groove to reconstruct the anterior and middle column through OLIF approach. When bone grafting, iliac bone was slightly higher than the height of intervertebral space or bone groove between the vertebrae. It helps provide sufficiently stable after grafting [29]. In this study, there was no incidence of internal fixation or bone graft failure in patients receiving this surgery. Bone fusion was seen in all these cases within 1 year after operation (Figs. 1H, 2H). The internal fixation device, served as a temporary fixation, were encouraged to be removed through the original minimal incision 1 year after surgery when bone fusion reached to Eck grade I [14]. Internal fixation removal was conducive to resuming the activity of adjacent normal segments, especially in young patients. There were 12 cases in group 1 and 6 cases in group 2 following the advice to remove the internal fixation, while other patients ignored the advice as they did not feel any discomfort.

As with all spine surgeries, delicate operations were required to avoid surgical damage. In freehand MIPS, carefully probe the pedicle bone canal in all four quadrants to ensure a solid bone tube exist to avoid pedicle screws intruding into the spinal canal or intervertebral foramen before pedicle screws implanted through minimal incision. In OLIF, structures covering TB lesion need to be clearly distinguished and dissected, such as aorta, iliac vessels, ureters, sympathetic nerve trunks. And operations on the surface of vertebrae are suggested peel along the subperiosteum [30]. In this study, there was no occurrence of neurologic complications or viscera and vascular injury in group 1. While in group 2 of posterior open surgery, there were 2 cases of dural sac tear, 3 cases of leg numb and hypoesthesia and 1 case of leg muscle weakness resulting from nerve root damage. No postoperative infection occurred. Drainage tubes were removed when the drainage volume was less than 20 ml/24 h. Sufficient drainage is an important measure to prevent postoperative infection.

This study still has some limitations. It was a retrospective study and conducted in a single medical center. And the number of patients receiving this surgery was relatively small. A prospective multicenter cohort study with larger samples will be performed to further confirm the clinical effects of this minimally invasive surgery in future. In addition, there is a lack of comparison with patients only performing chemotherapy. We will collect the patients only performing chemotherapy and further compare the fusion rate and sagittal parameters with those of surgery groups for the next study.

## Conclusion

One-stage freehand MIPS combined with mini-access surgery through OLIF approach is a feasible, efficient and safe method in treating single segment lumbar TB. It shows advantages of less surgical trauma and faster postoperative recovery.

## Data Availability

The raw data supporting the conclusions of this article will be made available by the authors, without undue reservation.
